# Migration of hem-o-lock clips and stitches into the duodenum after laparoscopic hepatectomy and cholecystectomy: A case report

**DOI:** 10.1097/MD.0000000000018153

**Published:** 2019-11-27

**Authors:** Yan Xia, Xiao-fei Gao, Cheng-yu Shi, Yuan-hui Jiang, Xin Yi

**Affiliations:** a Department of Pathology; b Department of Medical Imaging, Qilu Hospital of Shandong University (Qingdao); c Department of Hepatobiliary Surgery, The 2nd Affiliated Hospital of Medical College Qingdao University; d Department of General Surgery, Qilu Hospital of Shandong University (Qingdao), Qingdao, China.

**Keywords:** clip migration, duodenal obstruction, hem-o-lock clip, laparoscopic cholecystectomy, laparoscopic hepatectomy

## Abstract

**Rationale:**

Migration of endoclips and stitches into the duodenum after laparoscopic hepatectomy is incredibly rare with a poorly understood mechanism.

**Patient concerns:**

A 56-year-old woman who underwent laparoscopic left hepatectomy and cholecystectomy in August 2016 was admitted to our hospital with nausea and vomiting in December 2017.

**Diagnoses:**

Abdominal computed tomography (CT) scan showed high density shades in duodenal ampulla. Esophagogastroduodenoscopy showed deformation of the duodenal ampulla into two lumens; hem-o-lock clips and stitches were detected in the upper lumen. Contrast enhanced CT scan revealed gastric cancer with liver metastasis (GCLM).

**Interventions:**

The hem-o-lock clips and stitches were present in the wall of the duodenum; therefore, no attempt was made to remove them. High quality liquid diet, partial parenteral nutrition, and chemotherapy were administered to the patient.

**Outcomes:**

In September 2018, the patient died of hepatic failure caused by GCLM.

**Lessons:**

This rare complication of the migration of endoclips and stitches into the duodenum after laparoscopic hepatectomy can cause epigastric pain and duodenal obstruction. The complication could be potentially avoided using absorbable endoclips and stitches or by performing of ultrasonic dissection by a skilled operator.

## 1 Introduction

Laparoscopic hepatectomy and cholecystectomy are the currently preferred procedures for patients with hepatolithiasis and cholecystolithiasis, respectively, resulting in good aesthetic outcomes and rapid recovery. In these procedures, surgical clips and stitches are used laparoscopically to control bile duct, hepatic vessels, and cystic artery. However, wide use of surgical clips, such as hem-o-lock clips, in laparoscopic cholecystectomy has led to a variety of complications.^[1]^ Endoclip migration after laparoscopic cholecystectomy is a rare complication and can occur years after the procedure.^[2]^ A previous study reported that most clips migrated into common bile duct (CBD) and few migrated into the duodenum or other body parts.^[3]^ In this study, we present a rare case of migration of hem-o-lock clips and stitches into the duodenum after laparoscopic left hepatectomy and cholecystectomy.

The Ethics Committee of Qilu Hospital of Shandong University (Qingdao) (Grant No. KYLL-2018004, May 14, 2018) approved this study, and the written informed consent was obtained from the patient.

## 2 Case report

**Figure 1:**
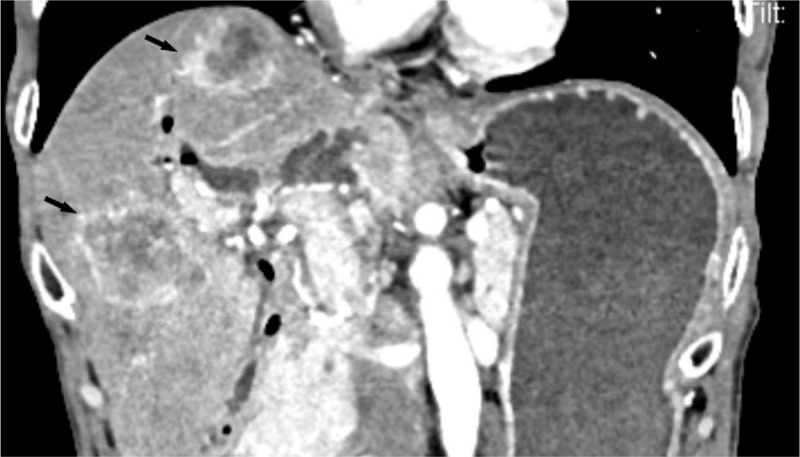
Contrast enhanced computed tomography (CT) scan showed hepatic metastasis (arrow).

**Figure 2:**
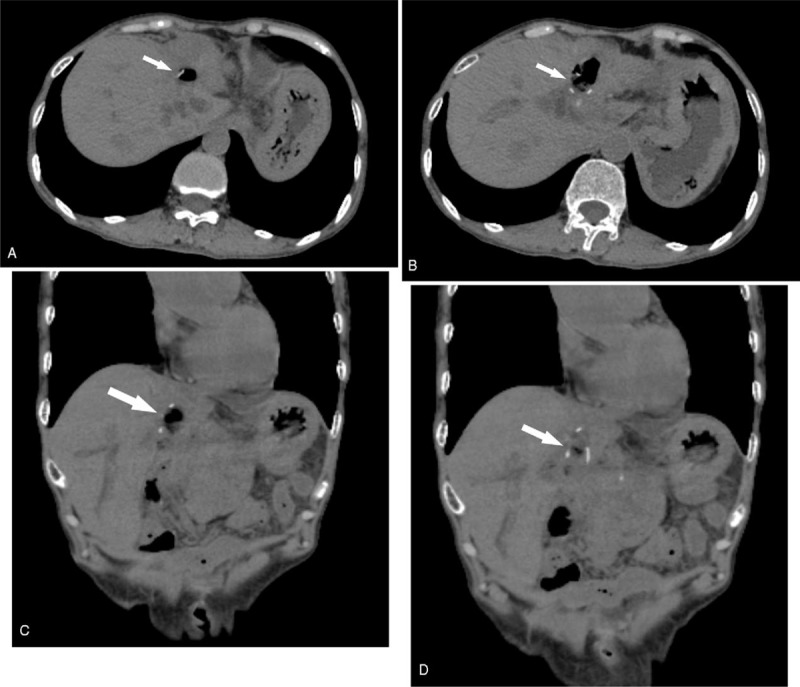
Computed tomography (CT) scan showed high density shades on the duodenal wall (arrow).

**Figure 3:**
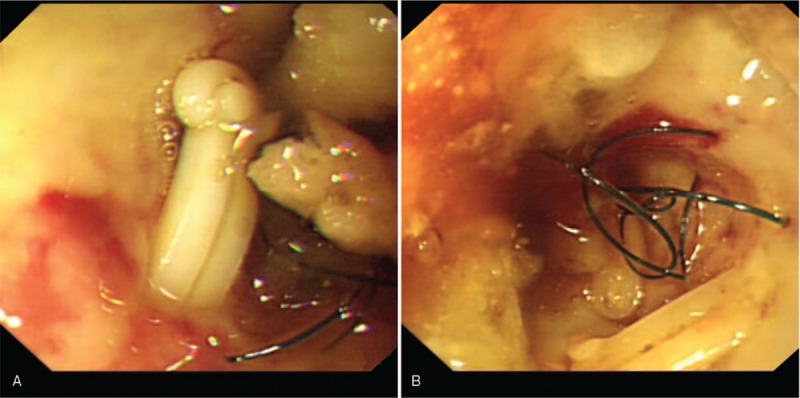
Endoscopic image of the duodenum showed hem-o-locks (A) and stitches (B) in the first part of duodenum wall.

A 56-year-old woman had epigastric pain in June 2016; her abdominal computed tomography (CT) scan showed gallbladder stones and hepatoliths. Laparoscopic left hepatectomy and cholecystectomy were performed in August 2016. Preoperative imaging showed normal extra-hepatic biliary anatomy (no short or long cystic ducts). One year later, the patient experienced epigastric pain which radiated to the back and nausea which worsened after eating. She lost 7-kg weight in 3 months. There was no history of gastrointestinal bleeding or jaundice. In December 2017, abdominal CT scan revealed gastric cancer with liver metastasis (GCLM) (Fig. 1). It also showed high density shades in the duodenal ampulla (Fig. 2). Esophagogastroduodenoscopy (EGD) showed deformation of the duodenal ampulla into two lumens; hem-o-lock clips and stitches were detected in the upper lumen, while the lower lumen was stenosed with compression (Fig. 3). The hem-o-lock clips and stitches were present in the wall of the duodenum; therefore, no attempt was made to remove them. Subsequently, CT-guided biopsy of the liver carcinoma was performed with the histopathological diagnosis being adenocarcinoma. Immunohistochemical analysis showed positivity for human epidermal growth factor receptor-2 (Her-2); further diagnosis of GCLM was made. The patient was subsequently transferred to the Department of Oncology for high quality liquid diet, partial parenteral nutrition, and chemotherapy. The patient died of hepatic failure caused by GCLM in September 2018.

## 3 Discussion

Complications associated with postcholecystectomy clip migration (PCCM) are rare.^[4]^ Most cases are related to migration into the CBD.^[1,4]^ The incorporation of clips into the duodenal wall is a rare occurrence. Clip migration into the duodenum was first reported in 1997.^[3]^ Less than fifteen such cases have previously been reported in the English medical literature.^[5]^ In all these cases, only laparoscopic cholecystectomy was performed, while in our case, the patient underwent laparoscopic left hepatectomy and cholecystectomy.

In previous cases, only metallic or hem-o-lock clips were found in the CBD or duodenum. However, in this case, apart from hem-o-lock clips, stitches were also found by EGD. In most reported cases, clips were usually found in duodenal ulcers with active bleeding.^[6]^ However, no evidence of ulcer or active bleeding was present in this case.

A variety of mechanisms of clip migration have been proposed.

(1) The first aspect to consider here is the procedure. Because of the incomplete closure of the cystic duct caused by ineffective clips, the duct remains partially patent resulting in bile leakage.^[7]^
(2) Anatomically, the duodenal wall lies adjacent to the ligated cystic duct and cystic artery stump; therefore, the clips may directly erode the duodenal wall.^[8]^ Moreover, the difficult dissection at the Calot's triangle and clip-induced inflammatory process may cause adhesion between the duodenum and ligated cystic duct and arterial stump with subsequent formation of choledochoduodenal fistula.^[8,9]^
(3) Despite the low rate of occurrence, an undiagnosed pre-existing duodenal ulcer may cause adherence of the duodenum to the gallbladder fossa, capturing the clip in the inflammatory process.^[6,10]^ However, none of these mechanisms was found to cause clip migration in our case.
(4) In the present study, the patient had GCLM. Normal anatomic relationship was destroyed by the growing tumor, which compressed the hem-o-lock clips and stitches into the duodenal wall.

Absorbable clips and stitches have been used more commonly since several years. Although the cost is marginally higher, absorbable clips and stitches should be the first choice to eliminate the possibility of clip migration.^[1,11]^ Ultrasonic dissection without the use of clips is also a viable option and could be broadly used in laparoscopic hepatectomy. Both the meticulous dissection of the Calot's triangle and the accurate placement of clips depend on the surgical skill of operators.^[12]^ Misplaced and wandering clips should be quickly detected and removed during the procedure. Comprehensive preoperative assessments such as CT, magnetic resonance imaging (MRI), magnetic resonance cholangiopancreatography (MRCP), are also important to identify anatomic variations.

## Author contributions

**Data curation:** Xiao-fei Gao, Cheng-yu Shi.

**Formal analysis:** Cheng-yu Shi, Yuan-hui Jiang.

**Resources:** Yuan-hui Jiang.

**Supervision:** Xin Yi.

**Writing – original draft:** Yan Xia.

**Writing – review & editing:** Yan Xia.
